# Widely targeted metabolomics reveals maturity-associated metabolic differences and their associations with style characteristics in flue-cured tobacco from distinct ecological regions of Guizhou

**DOI:** 10.3389/fpls.2026.1827811

**Published:** 2026-05-21

**Authors:** Chenyu Cai, Ao Gao, Xueyan Jing, Zeyu Zhao, Qi Xu, Qiong Liu, Kesu Wei, You Wu, Shuai He, Jun Jiang, Xiaodong Xie, Gaoyi Cao, Shengjiang Wu

**Affiliations:** 1 College of Agronomy and Resources and Environment, Tianjin Agricultural University, Tianjin, China; 2 Guizhou Academy of Tobacco Science, Upland Flue-Cured Tobacco Quality and Ecology Key Laboratory of China Tobacco, Guiyang, China; 3 Zunyi Tobacco Company of Guizhou Province, Zunyi, China; 4 Bijie Tobacco Company of Guizhou Province, Bijie, China; 5 Zhangjiachuan Hui Autonomous County Agricultural Technology Service Station, Tianshui, China

**Keywords:** enzyme activity, flue-cured tobacco, maturity, style characteristics, widely targeted metabolomics

## Abstract

To clarify maturity-associated metabolic differences in flue-cured tobacco grown in distinct ecological regions of Guizhou and their relationships with post-curing style characteristics, middle leaves of Yunyan 87 were collected from Dafang County (DF) and Weining County (WN), representing honey-sweet and fresh-sweet regions, respectively, at 16 (T1), 18 (T2), 20 (T3), 22 (T4), and 24 (T5) days after topping. Leaf phenotypes, color parameters, SPAD values, key enzyme activities, and sensory style scores were assessed across the five stages, whereas widely targeted metabolomics was performed on cured leaves from T1 and T5, resulting in four metabolomic groups: DF_T1, DF_T5, WN_T1, and WN_T5. Local environmental and soil variables and RT-qPCR validation of selected pathway genes were incorporated to strengthen ecological and transcriptional interpretation. With delayed harvest, leaves from both regions changed from green to yellow-green and pale yellow, the midrib gradually lost greenness and turned whitish, and trichome shedding increased; these phenotypes appeared earlier in DF. SPAD values generally declined in both regions and remained higher in WN than in DF. POD, LOX, PPO, and SPS activities increased with maturity, with generally higher activities in DF. Sensory evaluation indicated that the target style characteristics of cured leaves from both regions were enhanced with increasing maturity. Widely targeted metabolomics of cured leaves identified 72 differential metabolites in DF between T5 and T1, including 44 upregulated and 28 downregulated metabolites, with high proportions of flavonoids, alkaloids, terpenoids, and other phenolic-related metabolites. In WN cured leaves, 51 differential metabolites were identified, including 19 upregulated and 32 downregulated metabolites, mainly associated with sugar metabolism, glutathione metabolism, flavonoid biosynthesis, and ABC transporters. Environmental correlation analysis suggested that radiation-, temperature-, precipitation-, and soil-related variables were associated with region-specific flavonoid and antioxidant-related metabolic patterns. RT-qPCR of selected flavonoid biosynthesis and sugar metabolism/transport genes in fresh leaves supported pathway-level transcriptional divergence, although partial transcription–metabolite decoupling was also observed. Collectively, these results reveal regional differences in physiological and metabolic remodeling at key maturity stages and provide preliminary metabolomics-based evidence for optimizing harvest timing and style-oriented quality regulation of flue-cured tobacco in Guizhou.

## 1 Introduction

China is the world’s largest producer and consumer of tobacco (Zhu et al., 2024). Tobacco (*Nicotiana tabacum* L.) serves not only as a significant economic crop but also as a valuable model system for investigating plant secondary metabolism and responses to environmental stimuli (Sierro et al., 2014; Ma et al., 2019). In flue-cured tobacco, the final leaf quality and economic value are closely tied to field growth and development, with maturity representing a key determinant of appearance quality, chemical balance, and the full expression of varietal style characteristics (Wu et al., 2020; Wei et al., 2024; Yang et al., 2025b). During maturation, tobacco leaves undergo a series of complex physiological and biochemical transformations. The composition and abundance of metabolites continue to evolve, and variations in the chemical constituents of fresh leaves further influence aroma quality and style expression after curing (Wei et al., 2024).

The formation of tobacco style primarily results from the transition from primary to secondary metabolism, along with the subsequent thermal and chemical processes that occur during curing (Nugroho et al., 2002a). Under field conditions, topping redirects tobacco growth from vegetative development toward leaf maturation and metabolite accumulation. Primary metabolites such as amino acids, carbohydrates, and organic acids undergo continuous adjustment, whereas secondary metabolites including alkaloids, polyphenols, and terpenoids gradually accumulate (Nugroho et al., 2002b; Zhao et al., 2018; Shi et al., 2025). Concurrently, coordinated changes in plastid pigments, such as chlorophylls and carotenoids, may influence the formation of carotenoid-derived aroma precursors (Gerjets et al., 2007). The reallocation of carbon and nitrogen metabolism affects the conversion of starch to reducing sugars, the accumulation of amino acids, and the supply of substrates for subsequent Maillard reactions (Scheible et al., 1997). Furthermore, systematic remodeling of phenylpropanoid, terpenoid, and alkaloid metabolism can further modulate the abundance and composition of aroma precursors and their glycosidically bound reserves, thereby influencing their conversion during curing (El Tamer et al., 2003; Singh et al., 2025; Yang et al., 2025a). Therefore, the composition of metabolites and their variation at key stages of tobacco leaf maturation is essential for determining the optimal harvest time.

In addition to genetic regulation, tobacco secondary metabolism is jointly shaped by ecological conditions in the growing region and by curing practices (Chen et al., 2021; Shimasaki et al., 2021; Ye et al., 2021). Tobacco-growing areas in Guizhou are characterized by complex topography and diverse ecological types. Pronounced regional differences in altitude, temperature, solar radiation, precipitation, and soil nutrients can influence leaf maturation rates, metabolic phenotypes, and the final expression of sensory attributes (Lei et al., 2014; Zhao et al., 2016). Dafang (DF) and Weining (WN) are two representative tobacco-growing regions in Guizhou, characterized by honey-sweet and fresh-sweet style attributes, respectively. According to the national regionalization of flue-cured tobacco aroma styles, honey-sweet tobacco is characterized mainly by prominent honey-sweet and hay-like notes, whereas fresh-sweet tobacco is characterized mainly by fresh-sweet and green notes (Luo et al., 2019; Zhong et al., 2025). Comparing physiological, biochemical, and metabolic differences during leaf maturation in these two regions may enhance our understanding of the material basis underlying regional divergence in tobacco leaf style.

In recent years, analytical techniques such as GC-MS have been widely used to investigate volatile compounds associated with tobacco style (Likić, 2009), whereas metabolomics can reveal metabolite composition, abundance changes, and their biological significance at the systems level, offering clear advantages for studies of quality formation and maturation processes (Zhang et al., 2012; Qi and Zhang, 2014). Metabolomics has already been extensively applied to quality formation and maturation studies in horticultural crops such as bamboo shoots, apples, and carrots (Leiss et al., 2013; Gao et al., 2019; Xie et al., 2021). Among these approaches, widely targeted metabolomics combines broad coverage with robust quantitative performance and is well suited to systematically characterizing non-volatile metabolites in cured tobacco leaves derived from different harvest stages (Willency et al., 2024). Accordingly, this study used middle leaves of Yunyan 87 grown in Dafang and Weining and integrated phenotypic observation, color-parameter measurements, SPAD values, enzyme activities, sensory evaluation, widely targeted metabolomics of cured leaves, environmental correlation analysis, and RT-qPCR validation of fresh leaves to compare post-curing metabolic differences between two representative harvest stages and to explore their relationships with post-curing style characteristics. The results are expected to provide preliminary physiological and metabolic evidence for subsequent harvest-timing optimization and style-oriented quality regulation of flue-cured tobacco in Guizhou.

## 2 Materials and methods

### 2.1 Study sites and plant materials

Field experiments were conducted in 2024 at the Heishi Town Science and Technology Park in Weining County, Bijie City, Guizhou Province (a representative fresh-sweet-style tobacco region, mean altitude approximately 2200 m), and at the Liulong Town Science and Technology Park in Dafang County (a representative honey-sweet-style tobacco region, mean altitude approximately 1500 m). Sample detection and data analysis were completed during 2024-2025. The tested cultivar was Yunyan 87. Seedlings were transplanted on 28 April, with a row spacing of 120 cm and a plant spacing of 50 cm, and managed according to local high-quality flue-cured tobacco production practices. Middle leaves at the 10th leaf position were used as the experimental material and were tagged in the field. To support ecological interpretation, meteorological data during the 2024 growing season, including mean temperature, precipitation, rainy days, and solar radiation, were obtained from local meteorological or field-monitoring records. Soil physicochemical properties of the two sites were also summarized and used for environmental correlation analysis (**Supplementary Table 1**). Because direct UV-B measurements were not available for all batches, solar radiation was used as an approximate indicator of light exposure.

### 2.2 Experimental design and sample collection

Lower leaves without curing value (four leaves) were removed 1 week before topping. A preliminary reference harvest of four leaves was conducted 10 days after topping. The formal experiment included five sampling stages: T1 (16 days after topping), T2 (18 days), T3 (20 days), T4 (22 days), and T5 (24 days). The field was divided into 15 plots, and three replicate plots were randomly selected for each treatment; each plot covered 0.089 hm^2^. For each treatment, 30 tagged plants at the 10th leaf position were selected to investigate phenotypic traits and color parameters. After harvest, leaves from each treatment were tied to curing sticks, placed at the same position in the middle zone of the curing barn, and flue-cured using the Guizhou intensive-curing procedure in a downdraft, three-layer bulk curing barn (8.00 m x 2.80 m x 3.50 m, length x width x height). Thus, all samples were cured under the same barn type, position, and curing procedure to minimize variation caused by curing operations. In addition, fresh leaf samples were collected at T1 and T5, with the midrib and secondary veins removed, immediately frozen in liquid nitrogen, and stored at −80 °C for enzyme-activity assays and RT-qPCR validation. For metabolomic analysis, the corresponding cured tobacco leaves from T1 and T5 were collected after curing. These cured samples represented four treatment groups: DF_T1, DF_T5, WN_T1, and WN_T5. Six biological replicates were prepared for each treatment by generating two composite subsamples from each of the three field replicate plots.

### 2.3 Phenotypic observation and determination of color parameters

Changes in lamina color, degreening of the main midrib, and trichome shedding were recorded during maturation of tagged plants under each treatment, and representative leaf images were photographed under natural light. Color parameters were measured using a CR-10 colorimeter (Konica Minolta Sensing Inc., Japan) at symmetric positions approximately 5 cm away from the midrib (Wu et al., 2020). Three points were measured on each half leaf, for a total of six points per leaf, and the average value was used as the color parameter for that leaf. Ten leaves were measured for each treatment. The recorded color parameters were L*, a*, b*, C*, and H*, representing lightness, the red–green coordinate, the yellow–blue coordinate, chroma, and hue angle, respectively.

### 2.4 Determination of SPAD values and enzyme activities

Relative chlorophyll content was measured using a SPAD-502 meter (Konica Minolta Sensing Inc., Japan). Measurements were taken at symmetrical positions in the leaf tip, middle, and basal regions of each leaf, and the average of 15 leaves was used as the SPAD value for each treatment.

Commercial assay kits were purchased from Beijing Solarbio Science & Technology Co., Ltd. and used according to the manufacturer’s instructions to determine the activities of polyphenol oxidase (PPO; catalog no. QS1404), peroxidase (POD; catalog no. QS1502), sucrose-phosphate synthase (SPS; catalog no. QS2505), and lipoxygenase (LOX; catalog no. BC0320) (Anthon and Barrett, 2001; Broothaerts et al., 2000; Henry et al., 1974; Huber and Huber, 1991). Enzyme activity assays were performed with three biological replicates per treatment.

### 2.5 Sensory evaluation of post-curing style characteristics

For each treatment, 1.0 kg of cured tobacco leaves was collected for sensory evaluation according to YC/T 530-2015, Evaluation Method for Quality Style Characteristics of Flue-Cured Tobacco Leaves, and YC/T 138-1998, Sensory Evaluation Method for Tobacco and Tobacco Products. Seven trained panelists scored style dimensions including fresh-sweet and honey-sweet types. Style intensity was rated on a nine-point scale, and the mean score was used for analysis. In this study, the terms honey-sweet and fresh-sweet were not used as arbitrary descriptive labels. Honey-sweet style refers to a sensory profile dominated by honey-sweet and hay-like aroma notes, whereas fresh-sweet style refers to a profile dominated by fresh-sweet and green aroma notes, consistent with the national regionalization and sensory terminology for Chinese flue-cured tobacco aroma styles (Luo et al., 2019).

### 2.6 Widely targeted metabolomic analysis

Widely targeted metabolomic analysis was performed using cured tobacco leaf samples harvested at T1 and T5 from DF and WN. The four metabolomic groups were designated as DF_T1, DF_T5, WN_T1, and WN_T5. The metabolite extraction and LC–MS/MS analysis were performed with reference to a targeted metabolomics protocol and optimized for cured tobacco leaf samples (Willency et al., 2024). Briefly, 150 mg of sample was weighed into a 2 mL grinding tube, and 1 mL of pre-cooled extraction solvent (methanol:water = 7:3, v/v) was added. After addition of steel beads, samples were ground for 5 min at 50 Hz in a tissue grinder. Samples were then left to stand at 4 °C with intermittent vortexing and extracted overnight, followed by centrifugation at 13,000 g for 10 min at 4 °C. An 800 μL aliquot of the supernatant was collected and filtered through a 0.22 μm membrane prior to LC–MS/MS analysis.

LC–MS/MS analysis was performed using a Waters Acquity UPLC I-Class Plus system (Waters, USA) coupled to a QTRAP 6500 Plus mass spectrometer (SCIEX, USA). Separation was achieved on an HSS T3 column (2.1 mm × 10 cm, 1.8 μm; Waters). Mobile phase A consisted of water containing 0.1% formic acid, and mobile phase B consisted of acetonitrile containing 0.1% formic acid. The gradient program was as follows: 0–2.00 min, 5% B; 2.00–22.00 min, 5% B; 22.00–27.00 min, 95% B; 27.00–27.10 min, 95% B; and 27.10–30.00 min, 5% B. The flow rate was 0.300 mL min−1 and the column temperature was 40 °C. The ion-source temperature was 450 °C, the spray voltage was 5500 V in positive mode and −4500 V in negative mode, and GS1, GS2, and CUR were set to 40, 40, and 20 psi, respectively. Metabolite signals were acquired in multiple reaction monitoring (MRM) mode.

Raw data were processed using Skyline v21.1.0.146, and metabolites were annotated and quantified by matching against the BGI-Widetarget-Library. In Meta X, features with a missing rate >50% in quality-control samples or >80% in study samples were removed. Remaining missing values were imputed using the KNN method, data were normalized using probabilistic quotient normalization (PQN), batch effects were corrected by QC-RLSC, and features with a coefficient of variation >30% were excluded. One QC sample was inserted after every three study samples, and system stability was evaluated by PCA and QC-sample correlation analysis. PCA and OPLS-DA were performed in SIMCA 14.1, and 200 permutation tests were conducted. Differential metabolites were screened using the combined criteria of VIP ≥ 1, P < 0.05 from univariate statistical testing, and FC ≥ 1.2 or FC ≤ 0.83. VIP ≥ 1 was used to identify variables contributing substantially to group separation in the OPLS-DA model, the P-value criterion was used to retain metabolites with nominal statistical significance in pairwise univariate comparisons, and the FC threshold was used to ensure a minimum magnitude of biological change in the relative-abundance data. The combined use of multivariate contribution, univariate significance, and fold-change thresholds was intended to reduce false positives caused by relying on a single criterion. Metabolic pathway annotation was conducted using the KEGG database (https://www.kegg.jp/). Because the metabolomic data were based on relative quantification, representative key metabolites were interpreted as candidate markers unless independently validated by targeted absolute quantification.

### 2.7 RT-qPCR validation of key pathway genes

To provide transcriptional support for the pathway divergence suggested by the cured-leaf metabolomic analysis, RT-qPCR was performed using fresh leaf samples collected at the corresponding representative harvest stage. Because RT-qPCR reflects active transcriptional regulation in living tissues, fresh leaves were used for transcriptional validation. Cured leaves were not used for RT-qPCR because curing involves dehydration, heat treatment, enzymatic inactivation, and extensive biochemical and chemical transformations, which may reduce RNA integrity and no longer reliably reflect the transcriptional state of metabolic pathways during field maturation. Total RNA was extracted using the FastPure Universal Plant Total RNA Isolation Kit (Vazyme, Cat. RC411-01), and first-strand cDNA was synthesized using HiScript III RT SuperMix for qPCR (Vazyme, Cat. R323-00). Quantitative PCR was performed using 2× SuperStar qPCR Mix (CWBIO, Cat. CW3360) according to the manufacturer’s instructions. Seven candidate genes were analyzed, including *NtCHS*, *NtCHI*, *NtF3H*, *NtF3’H*, *NtSPS5*, *NtSPS6*, and *NtSWEET12i*. *NtEF-1alpha* and *NtL25* were used as internal reference genes. Relative expression levels were calculated using the 2^−ΔΔCt^ method, and statistical significance was assessed using Welch’s t-test based on ΔCt values. Primer sequences are provided in **Supplementary Table 2**.

### 2.8 Statistical analysis

Microsoft Excel 2021 and R were used for data processing and statistical analyses. Figures were prepared using GraphPad Prism 9.5 and Adobe Illustrator 2024. Phenotypic traits, color parameters, SPAD values, enzyme activities, and sensory evaluation data were analyzed by two-way ANOVA, followed by Duncan’s multiple range comparisons (P < 0.05). Metabolomic data were subjected to PCA and OPLS-DA, and differential metabolites were identified using the combined VIP, univariate P-value, and fold-change thresholds described above. Relationships among environmental variables, metabolites, and physico-biochemical traits were visualized by Pearson or Spearman correlation heatmaps and Mantel tests where appropriate. For RT-qPCR, relative expression levels were calculated from three biological replicates and compared using Welch’s t-test.

## 3 Results

### 3.1 Phenotypic and color-parameter changes in tobacco leaves during maturation in different ecological regions

**Figure 1:**
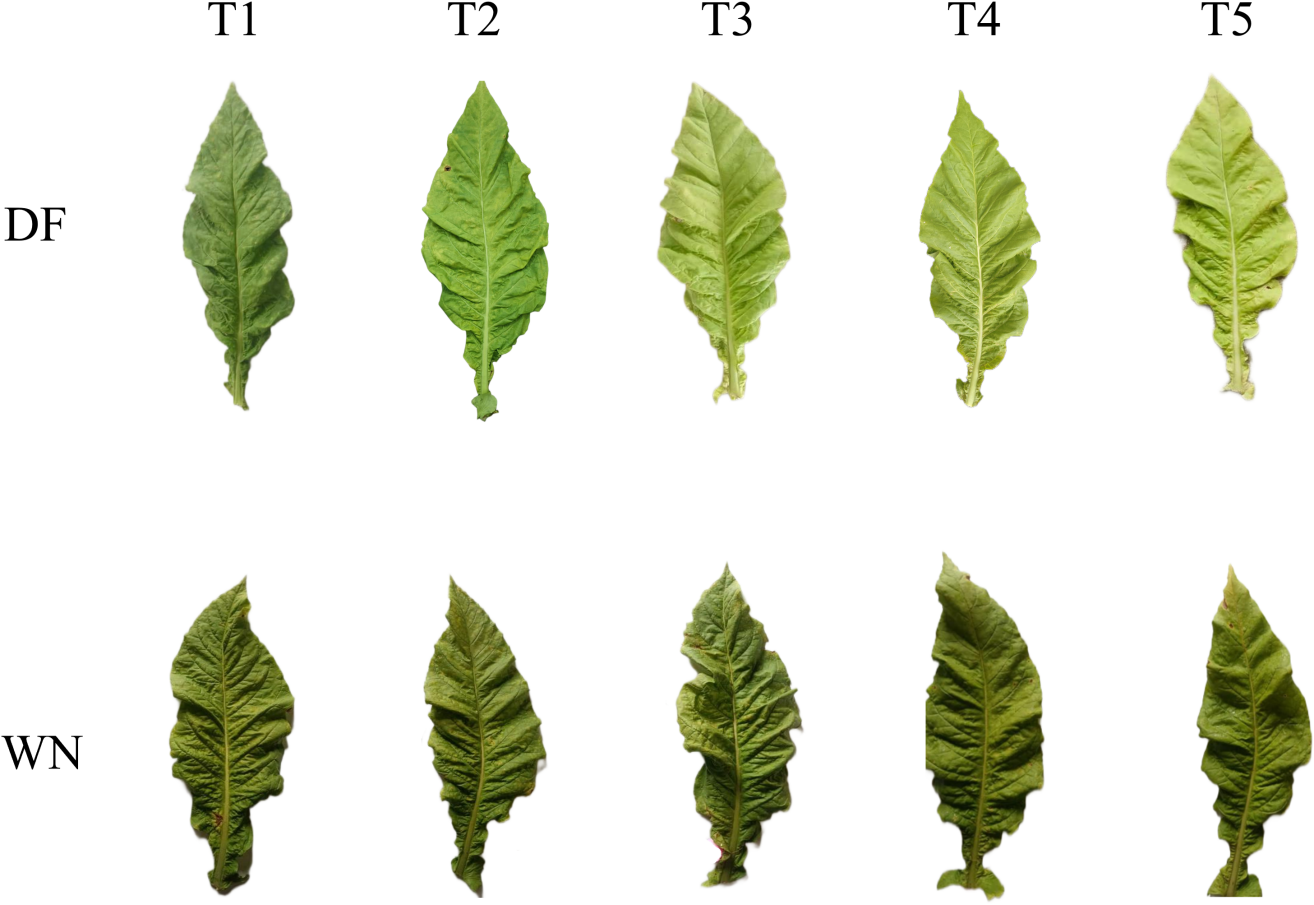
Phenotypic changes in tobacco leaves during maturation in different ecological regions.

To clarify changes in external phenotype and color characteristics during tobacco leaf maturation in different ecological regions, leaves from DF and WN were continuously observed from T1 to T5. The results showed that, with delayed harvest, leaves in both regions gradually changed from green to yellow-green and pale yellow, the main midrib progressively lost greenness and turned whitish, and trichome shedding increased, all of which are characteristic maturity-associated phenotypes (**Figure 1**). Overall, leaves from DF yellowed more rapidly and displayed more pronounced color changes, whereas leaves from WN yellowed more slowly and showed smaller changes in overall color, indicating clear differences in the rate of maturation between the two regions.

**Figure 2:**
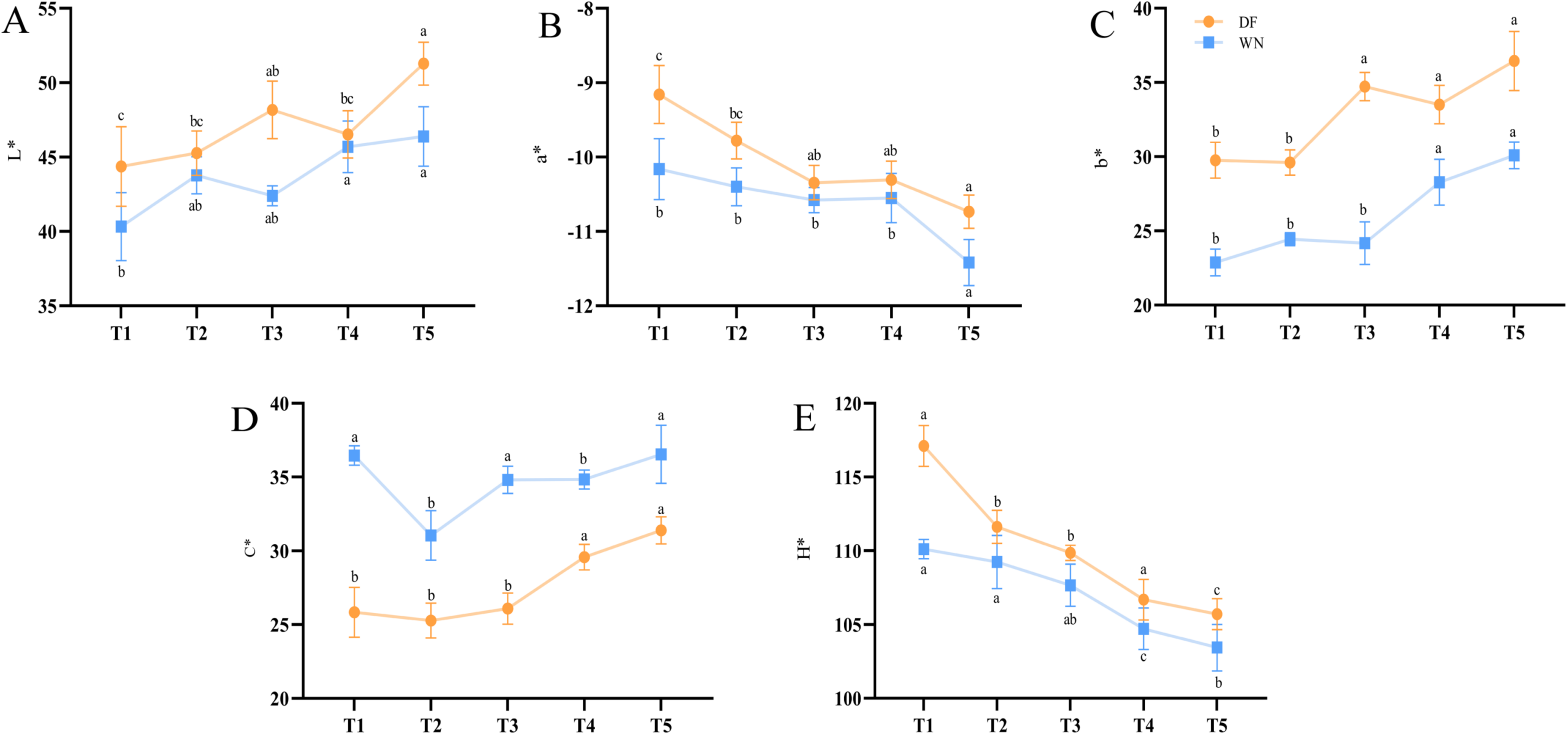
Changes in color parameters of tobacco leaves during maturation in different ecological regions. **(A)** Lightness (L*), **(B)** red–green coordinate (a*), **(C)** yellow–blue coordinate (b*), **(D)** chroma (C*), and **(E)** hue angle (H*). Values are presented as mean ± SD (n = 10 biological replicates). Different lowercase letters indicate significant differences at *p* < 0.05 based on Duncan’s multiple range test.

Color-parameter analysis further confirmed the phenotypic observations. With increasing maturity, L*, b*, and C* values generally increased in both DF and WN, whereas a* and H* values generally decreased (**Figure 2**), indicating progressive increases in brightness and yellowness together with a reduction in greenness. At the same sampling stage, DF leaves showed more pronounced changes in overall color, suggesting faster maturation, whereas WN leaves exhibited more gradual color changes, indicating relatively delayed maturation.

### 3.2 Changes in SPAD values and enzyme activities during tobacco leaf maturation in different ecological regions

**Figure 3:**
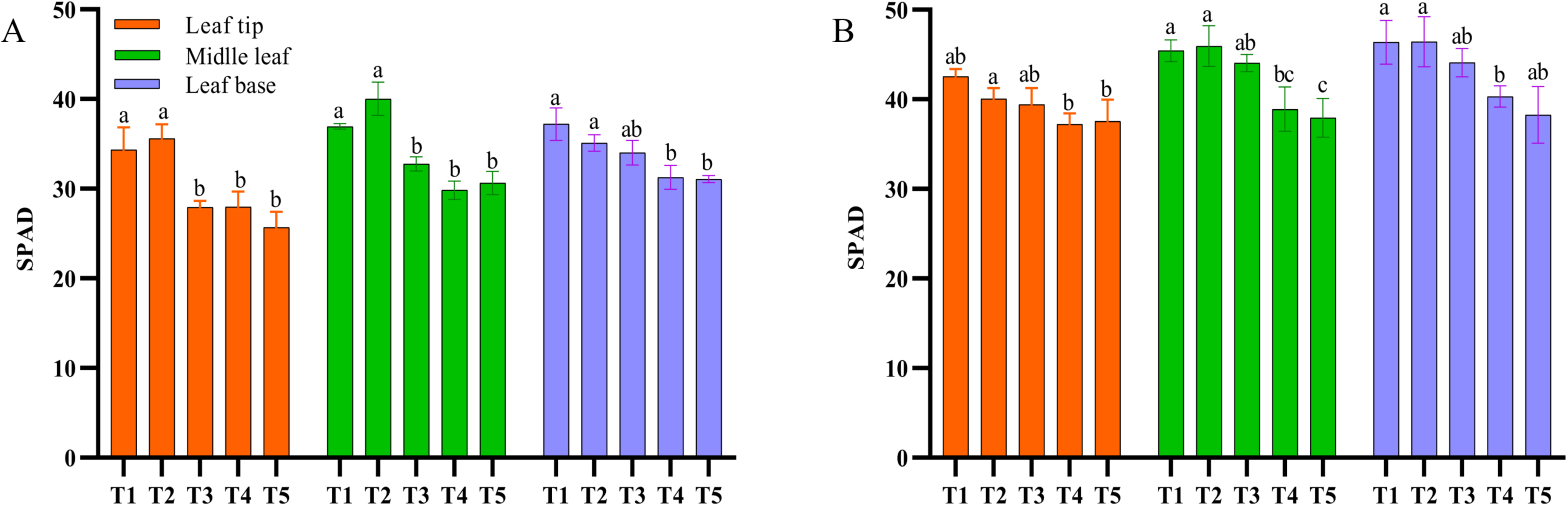
Changes in SPAD values of tobacco leaves during maturation in different ecological regions. **(A)** SPAD values of tobacco leaves during maturation in the DF region; **(B)** SPAD values of tobacco leaves during maturation in the WN region. SPAD values were measured at the leaf tip (upper part of the leaf), leaf middle (middle part of the leaf), and leaf base (basal part of the leaf). Values are presented as mean ± SD (n = 15 biological replicates). Different lowercase letters indicate significant differences at *p* < 0.05 according to Duncan’s multiple range test.

Relative chlorophyll content differed markedly among leaf regions, with the basal part generally showing higher SPAD values than the middle and tip regions (**Figure 3**). As harvest was delayed, SPAD values in all leaf regions generally declined, indicating gradual chlorophyll degradation during maturation. A regional comparison showed that WN leaves had consistently higher SPAD values than DF leaves, suggesting that chlorophyll degradation progressed more slowly in WN and that leaf maturation was correspondingly delayed.

**Figure 4:**
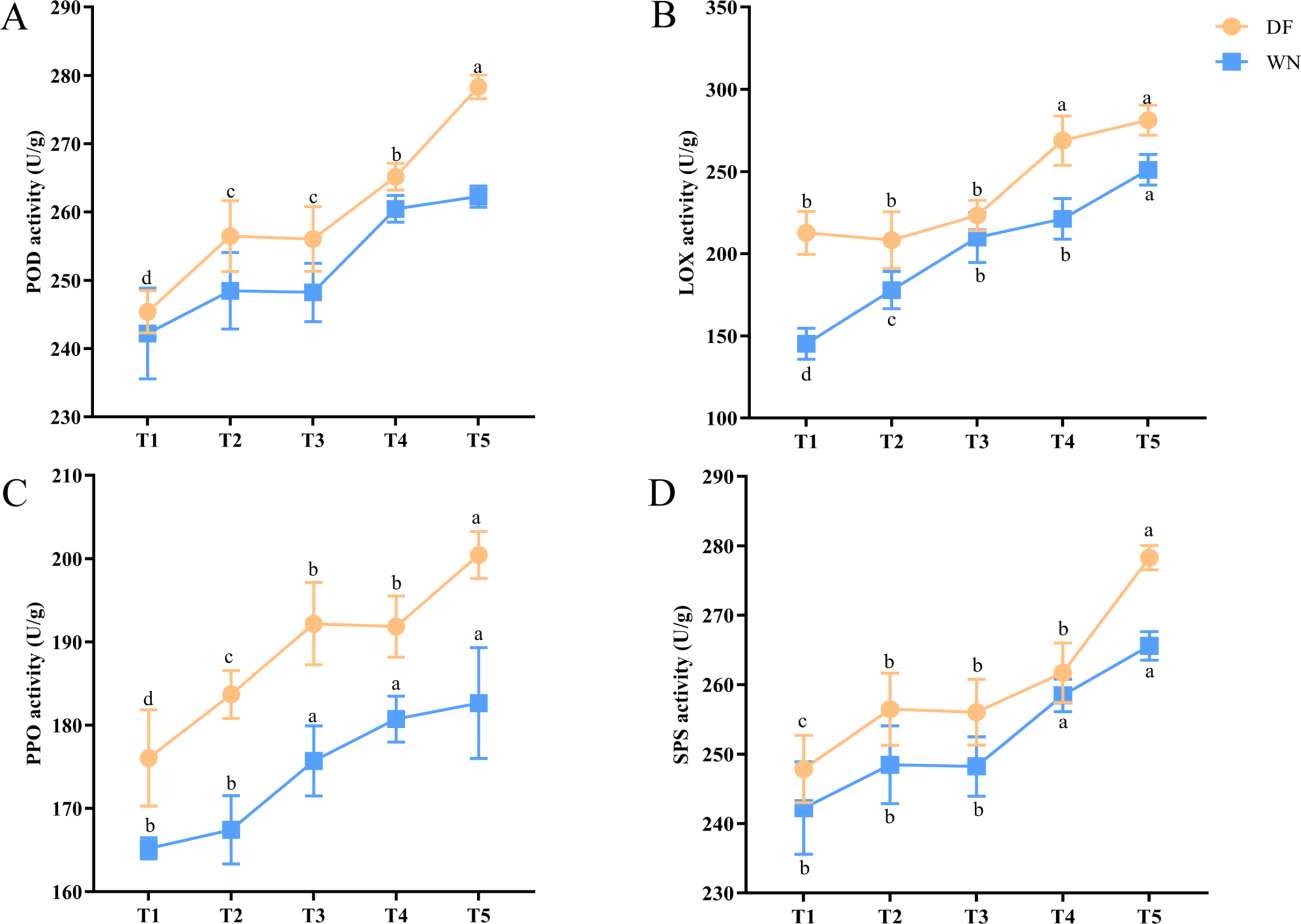
Changes in the activities of key enzymes in tobacco leaves during maturation in different ecological regions. **(A)** POD, **(B)** LOX, **(C)** PPO, and **(D)** SPS. Values are presented as mean ± SD (n = 3 biological replicates). Different lowercase letters indicate significant differences at *p* < 0.05 according to Duncan’s multiple range test.

The activities of four key enzymes generally increased during maturation (**Figure 4**). POD activity rose progressively from T1 to T5 and reached a relatively high level at T5. LOX activity increased in both DF and WN, although the patterns differed between regions. PPO activity continuously increased as maturity progressed, whereas SPS activity rose markedly during late maturation. Overall, enzyme activities were higher in DF than in WN at most sampling stages, indicating that physiological processes related to oxidative metabolism, sugar metabolism, and membrane-lipid metabolism were more active during maturation in DF leaves.

### 3.3 Sensory evaluation of post-curing style characteristics

**Figure 5:**
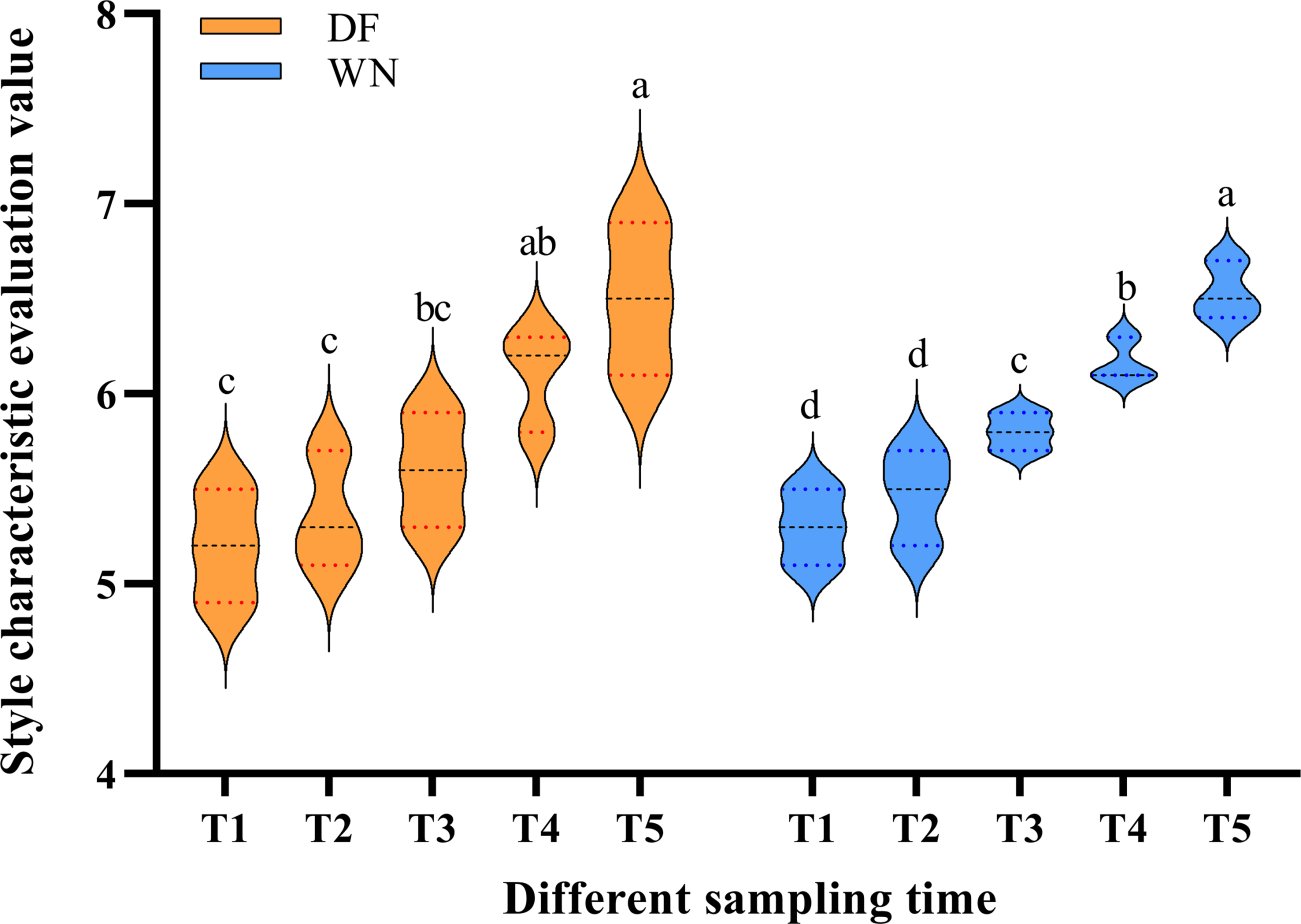
Violin plots of post-curing style characteristics of cured tobacco leaves harvested at different maturity stages. Different lowercase letters indicate significant differences among treatments at p < 0.05 according to Duncan’s multiple range test.

Sensory evaluation was performed for cured tobacco leaves harvested at different stages in the two regions based on different style dimensions and a nine-point style-intensity scale. The results showed that, with increasing maturity, scores for the honey-sweet style in DF and the fresh-sweet style in WN both tended to increase (**Figure 5**), indicating that a moderate delay in harvest favored the expression of the target style characteristics. This trend was generally consistent with the observed changes in phenotype, SPAD values, and enzyme activities, and provided a basis for further analysis of the metabolic basis underlying style differences.

### 3.4 Metabolomic analysis of cured tobacco leaves harvested at representative maturity stages

#### 3.4.1 PCA and OPLS-DA analyses

**Figure 6:**
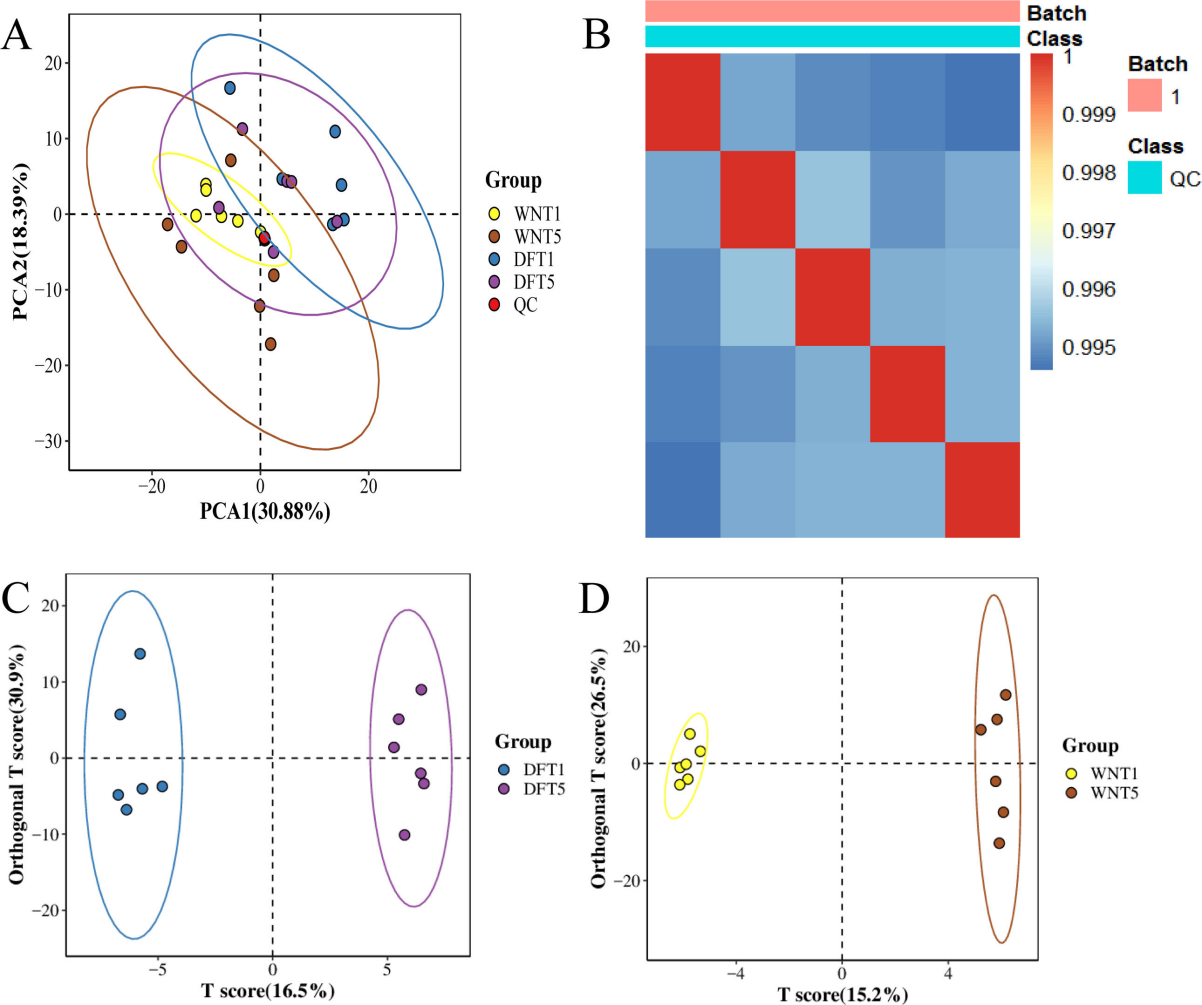
PCA and OPLS-DA analyses of metabolite profiles in cured tobacco leaves from different ecological regions and harvest stages. **(A)** PCA score plot for all samples. **(B)** Correlation heatmap of QC samples. **(C)** OPLS-DA score plot for the DF region. **(D)** OPLS-DA score plot for the WN region.

Based on differences in color parameters, enzyme activities, and post-curing style characteristics across the five harvest stages, cured tobacco leaves from T1 and T5 were selected for widely targeted metabolomic analysis. Four groups were included: DF_T1, DF_T5, WN_T1, and WN_T5, representing cured leaves harvested at the early and late maturity stages from the two ecological regions (**Figure 6A**). The first two principal components explained 30.88% and 18.39% of the total variance, respectively. Although the cumulative variance explained by the first two components was moderate, which is common for complex metabolomic datasets with diverse chemical classes, the distribution pattern still indicated major differences in metabolite composition between ecological regions and maturity stages. QC samples clustered tightly in PCA space, and the Spearman correlation coefficient reached 0.91, indicating stable instrument performance and good data reproducibility (**Figure 6B**). OPLS-DA likewise clearly separated T1 from T5 samples in both DF and WN (**Figures 6C, D**), demonstrating substantial metabolomic remodeling before and after maturation.

#### 3.4.2 Differential metabolite analysis

**Figure 7:**
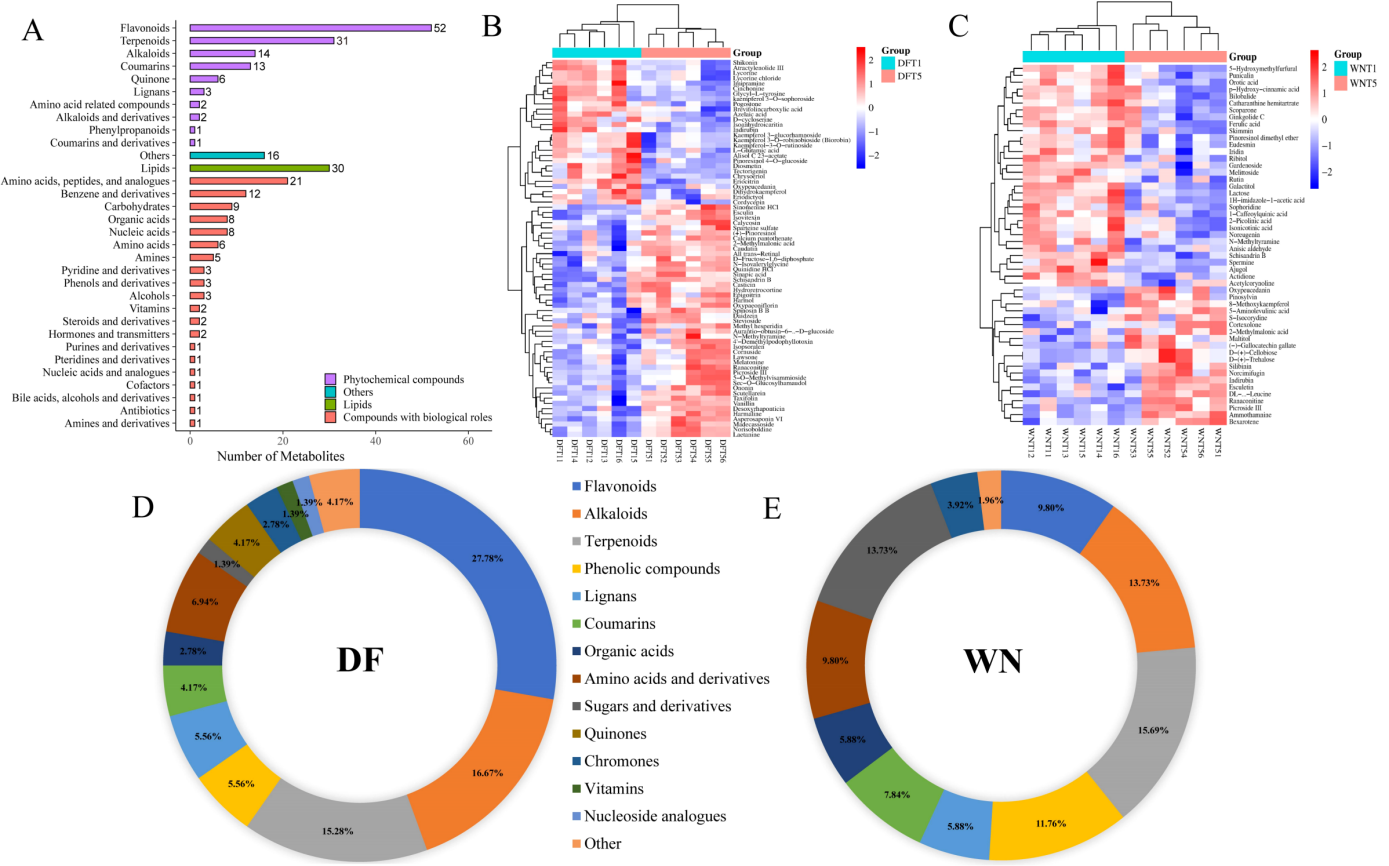
Differential metabolites in cured tobacco leaves from different ecological regions and harvest stages. **(A)** Numbers and chemical classes of differential metabolites in DF and WN. **(B)** Hierarchical clustering heatmap of differential metabolites in DF_T5 vs. DF_T1. **(C)** Hierarchical clustering heatmap of differential metabolites in WN_T5 vs. WN_T1. **(D)** Distribution of differential metabolites among metabolite classes in DF. **(E)** Distribution of differential metabolites among metabolite classes in WN.

Differential metabolites were identified for the two comparison groups, DF and WN, based on VIP values, P values, and fold-change criteria (**Figure 7**; **Supplementary Table 3**). In the DF_T5 vs. DF_T1 comparison of cured leaves, a total of 72 differential metabolites were identified, including flavonoids (27.78%), alkaloids (16.67%), terpenoids (15.28%), amino acids and derivatives (6.94%), phenolic compounds (5.56%), lignans (5.56%), coumarins (4.17%), quinones (4.17%), organic acids (2.78%), chromones (2.78%), sugars and derivatives (1.39%), vitamins (1.39%), nucleoside analogues (1.39%), and other compounds (4.17%). Among these, 44 metabolites were upregulated and 28 were downregulated. In the WN_T5 vs. WN_T1 comparison of cured leaves, a total of 51 differential metabolites were identified, including terpenoids (15.69%), alkaloids (13.73%), sugars and derivatives (13.73%), phenolic compounds (11.76%), flavonoids (9.80%), amino acids and derivatives (9.80%), coumarins (7.84%), lignans (5.88%), organic acids (5.88%), chromones (3.92%), and other compounds (1.96%). Among these, 19 metabolites were upregulated and 32 were downregulated. A hierarchical clustering heatmap was added to show the abundance patterns and clustering relationships of differential metabolites across samples (**Figure 7B**). Overall, differential metabolites in both regions were mainly represented by flavonoid/phenolic-related compounds, alkaloids, terpenoids, and amino acid-related metabolites, but their compositional profiles differed markedly between regions. Compared with WN, DF exhibited a substantially higher proportion of flavonoids among maturity-responsive differential metabolites, whereas WN showed relatively higher proportions of sugars and derivatives as well as phenolic compounds. These results indicate that harvest-stage-associated post-curing metabolic differences occurred in both regions, but the dominant metabolite classes and the direction of metabolic change differed between DF and WN (**Supplementary Table 4**).

#### 3.4.3 Pathway enrichment analysis of differential metabolites

**Figure 8:**
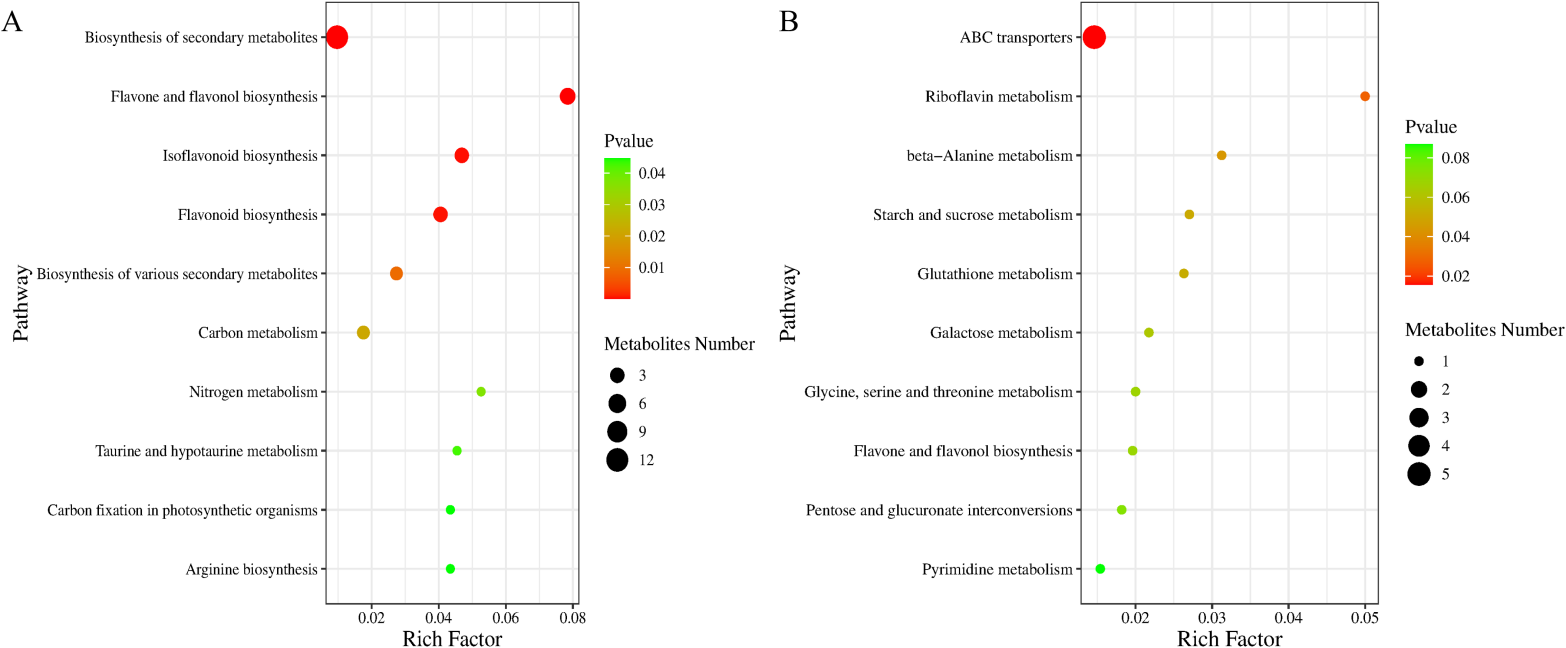
Bubble plots of enriched metabolic pathways of differential metabolites in cured tobacco leaves from different ecological regions. **(A)** Enriched metabolic pathways in DF. **(B)** Enriched metabolic pathways in WN.

KEGG enrichment analysis showed that, in the DF_T5 vs. DF_T1 comparison of cured leaves, differential metabolites were mainly enriched in secondary metabolite biosynthesis, flavone and flavonol biosynthesis, isoflavonoid biosynthesis, and flavonoid biosynthesis (**Figure 8A**). This enrichment pattern was consistent with the relatively high proportions of flavonoids and other phenolic-related metabolites observed in DF. Among the differential metabolites, cynaroside, diosmetin, daidzein, calycosin, ononin, atractylenolide III, oxypeucedanin, stevioside, sinapic acid, pinoresinol, and vanillin changed markedly, suggesting that late maturation in DF was closely associated with enhanced secondary metabolism.

In the WN_T5 vs. WN_T1 comparison of cured leaves, differential metabolites were mainly enriched in ABC transporters, flavonoid biosynthesis, riboflavin metabolism, beta-alanine metabolism, starch and sucrose metabolism, and glutathione metabolism (**Figure 8B**). This result was in agreement with the relatively higher proportions of sugars and derivatives, phenolic compounds, and antioxidant-related metabolites in WN. Trehalose, cellobiose, maltitol, and other sugars or sugar alcohols were markedly upregulated, and some flavonoid-related metabolites also changed significantly, indicating that late maturation in WN was characterized by stronger regulation of sugar metabolism, material transport, and antioxidant-related metabolism.

#### 3.4.4 Representative differential metabolites associated with post-curing style characteristics

**Figure 9:**
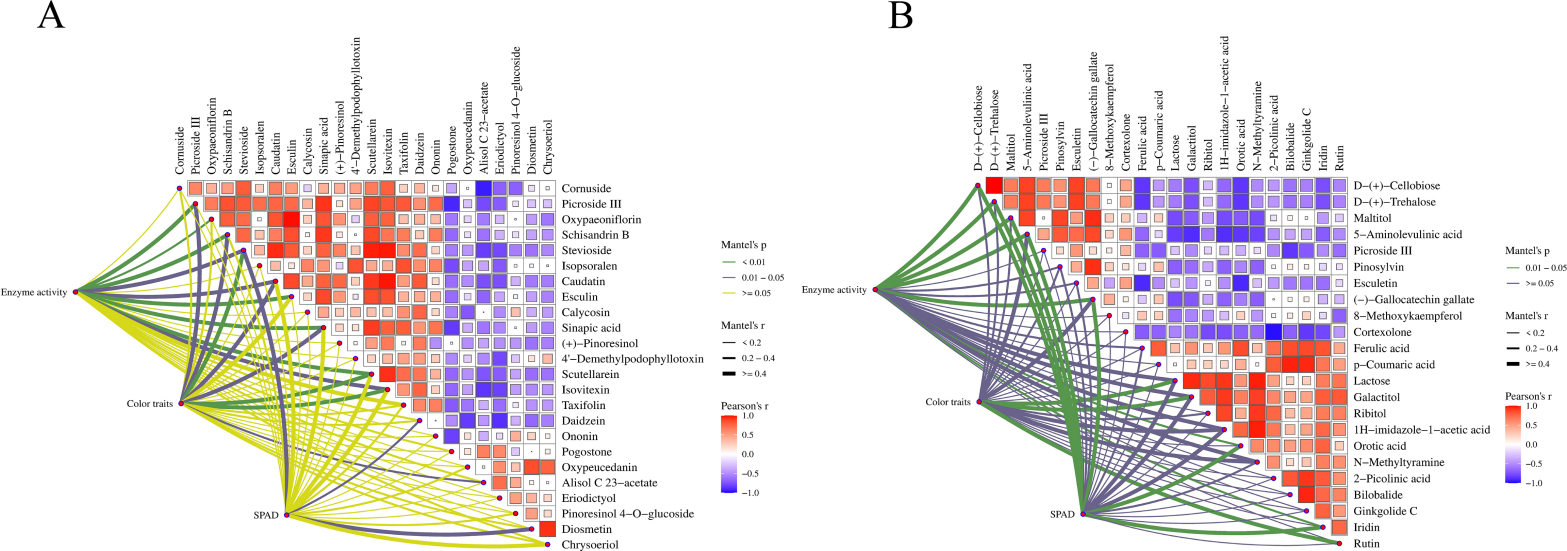
Correlation and Mantel-test network analysis of representative metabolites associated with post-curing style characteristics in tobacco leaves from different ecological regions. **(A)** DF region. **(B)** WN region. Line width and color indicate Mantel’s r and significance level, respectively.

Sensory evaluation showed that, with increasing maturity, the honey-sweet style in the DF region and the fresh-sweet style in the WN region were both enhanced. Combined with the metabolomics results, the representative differential metabolites upregulated during the late maturity stage in DF mainly included vanillin, sinapic acid, pinoresinol, and some flavonoid-, lignan-, and terpenoid-related metabolites (**Figure 9A**), whereas those upregulated in WN mainly included trehalose, cellobiose, maltitol, as well as some flavonoid- and antioxidant-related metabolites (**Figure 9B**). These metabolites were associated with the enhancement of the target post-curing style characteristics in the two regions. Because the present metabolomic analysis was conducted on cured leaves and based on relative quantification, these metabolites should be regarded as post-curing associated markers rather than direct causal determinants of final sensory style. Their abundance may reflect both field-maturation status and biochemical or chemical transformations occurring during curing.

### 3.5 Environmental correlations and RT-qPCR validation of pathway divergence

**Figure 10:**
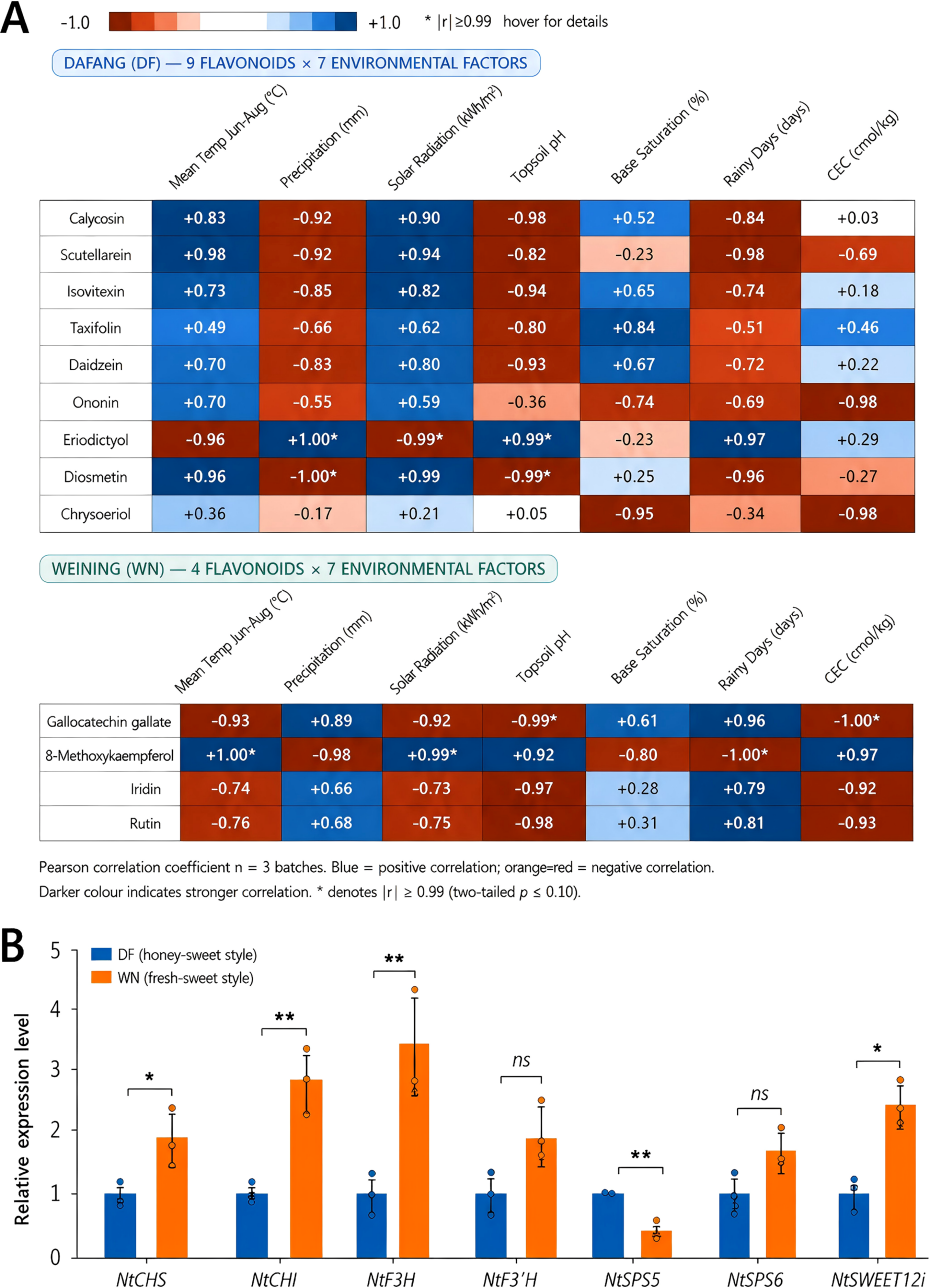
Environmental correlation and RT-qPCR validation of pathway divergence between DF and WN. **(A)** Exploratory Pearson correlation analysis between representative differential metabolites detected in cured leaves and environmental or soil variables in DF and WN. Blue indicates positive correlations, whereas orange-red indicates negative correlations; darker colors indicate stronger correlations. Asterisks indicate correlations with |r| ≥ 0.99. Correlations were calculated based on three environmental batches. **(B)** Relative expression levels of selected genes involved in flavonoid biosynthesis and sugar metabolism/transport in fresh leaf samples. DF represents honey-sweet-style tobacco, and WN represents fresh-sweet-style tobacco. Values are presented as mean ± SD (n = 3 biological replicates). Asterisks indicate significant differences between DF and WN (*P < 0.05; **P < 0.01); ns indicates no significant difference.

To strengthen the ecological interpretation of regional post-curing metabolic differences, environmental and soil variables from the two sites were integrated with representative differential metabolites detected in cured leaves (**Supplementary Table 1**; **Figure 10A**). Correlation analysis suggested that the abundance of representative flavonoid- and antioxidant-related metabolites in cured leaves was associated with temperature, radiation-related variables, precipitation-related variables, and soil properties in a compound-specific manner. These relationships support the possibility that ecological gradients during field growth contribute to region-specific post-curing metabolite profiles, although multi-year and multi-site validation is still required.

RT-qPCR analysis of fresh leaf samples further provided transcriptional support for pathway divergence during field maturation, which may partly underlie the post-curing metabolite differences detected by metabolomics (**Figure 10B**). For flavonoid biosynthesis, *NtCHS*, *NtCHI*, and *NtF3H* showed higher expression in WN than in DF, whereas *NtF3’H* showed a similar increasing tendency. For sugar metabolism and transport, *NtSWEET12i* was significantly more highly expressed in WN, *NtSPS5* showed higher expression in DF, and *NtSPS6* tended to be upregulated in WN. These results support the presence of divergent regulation in flavonoid biosynthesis and sugar metabolism/transport between the two regions. Meanwhile, the fact that gene-expression patterns were not identical to differential-metabolite proportions indicates partial transcription-metabolite decoupling and suggests that pathway regulation during tobacco maturation involves multiple levels, including transcription, enzyme activity, metabolite turnover, and environmental modulation.

## 4 Discussion

### 4.1 Differences in leaf phenotype and maturation progression between ecological regions

Tobacco leaf style is jointly influenced by internal metabolic status, ecological factors, and agronomic regulation, and its material basis is closely linked to the dynamic changes in metabolites during maturation and senescence (Bussotti, 2008). Changes in leaf morphology and color directly reflect the internal physiological and metabolic status and are particularly associated with pigment metabolism, leaf senescence, and redox regulation (Mendes-Pinto, 2009; Woo et al., 2019; Din et al., 2025). In this study, leaves from DF and WN exhibited typical maturity-associated traits as maturation progressed, including yellowing, whitening of the midrib, and trichome shedding; however, maturation advanced at different rates in the two regions. Based on color parameter changes, the more rapid external maturation of DF leaves may be associated with more active pigment degradation and conversion, whereas WN leaves likely undergo a more gradual maturation process. The differences observed in yellowing rate and overall color transition between the two regions indicate clear ecological differentiation in leaf-color change during maturation, consistent with plastid pigment remodeling as a key driver of leaf color change during tobacco maturation and curing (Wu et al., 2020).

### 4.2 Differences in SPAD values and enzyme activities during tobacco leaf maturation in different ecological regions

As tobacco maturity increased, SPAD values decreased continuously, indicating gradual chlorophyll degradation during maturation, which is an important physiological indicator of external leaf maturity. In this study, SPAD values were consistently higher in WN than in DF, suggesting slower chlorophyll degradation and delayed maturation in WN (**Figure 3**). This does not contradict the enhancement of the fresh-sweet style in WN, because SPAD reflects chlorophyll-retention status rather than serving as a direct flavor index. The fresh-sweet style in WN may be more closely associated with sugar metabolism, sugar transport, and antioxidant homeostasis than with rapid pigment degradation alone. This result is also consistent with previous findings showing that plastid pigment metabolism contributes to tobacco leaf color changes during curing and that maturity-related physiological markers may vary among ecological regions and cultivars (Wu et al., 2020; Wei et al., 2024; Yang et al., 2025b). Meanwhile, POD, LOX, PPO, and SPS activities all increased overall, indicating progressively enhanced processes related to redox regulation, membrane-lipid metabolism, phenolic oxidation, and sugar metabolism during maturation. LOX variation is typically associated with lipid oxidation and stress responses (Ali and Baek, 2020), SPS variation with sucrose synthesis and carbon-metabolism regulation (Scheible et al., 1997), whereas POD and PPO variation is often associated with phenolic oxidation and antioxidant defense (Ferreyra et al., 2021). These enzymes may contribute indirectly to the supply and transformation of aroma-related precursors during curing, but the present data support potential associations rather than direct causal links between enzyme activity and honey-sweet or fresh-sweet style formation.

### 4.3 Differences in metabolic remodeling patterns during late maturation in tobacco leaves from different ecological regions

**Figure 11:**
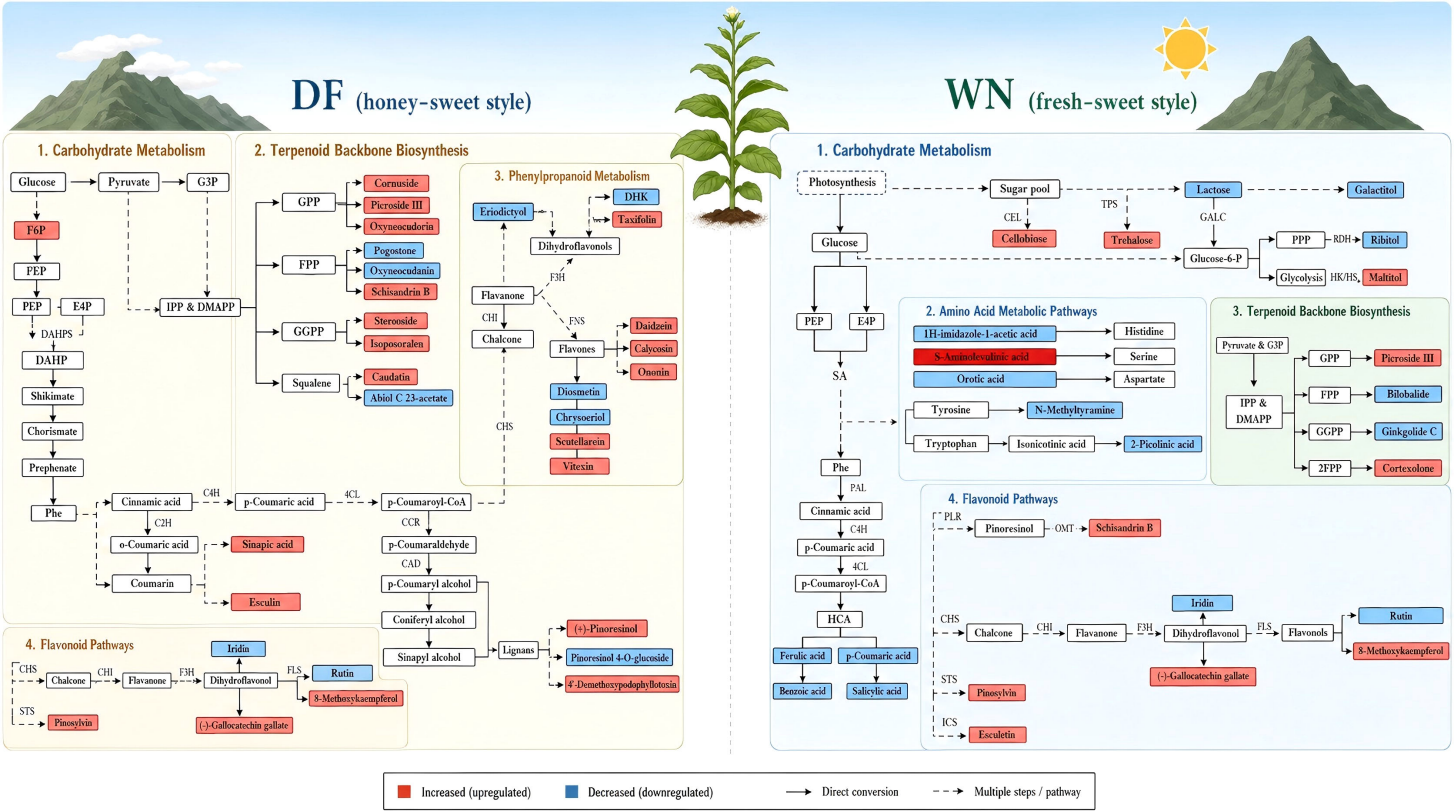
Proposed schematic model of region-specific metabolic remodeling during late maturation of flue-cured tobacco leaves. Dominant pathways in DF and WN are highlighted separately to emphasize differences in secondary metabolism, sugar metabolism/transport, and antioxidant-related metabolism.

Widely targeted metabolomics of cured leaves revealed that DF exhibited a greater number of differential metabolites associated with late-harvest/post-curing metabolic profiles, with prominent changes in flavonoids, alkaloids, terpenoids, and other phenolic-related metabolites, and with major enrichment in flavonoid biosynthesis and secondary metabolite biosynthesis pathways. Because the term biosynthesis of secondary metabolites represents a broad KEGG category, we further interpreted the results by focusing on more specific functional nodes, including flavone and flavonol biosynthesis, isoflavonoid biosynthesis, phenylpropanoid-related metabolites, and terpenoid-related metabolites (**Figures 8**, **11**). These more specific pathways are biologically relevant to tobacco maturation because phenylpropanoid and flavonoid metabolism is related to antioxidant protection, cell-wall modification, and aroma-precursor storage, whereas terpenoid metabolism is closely linked to the formation of aroma-related compounds (El Tamer et al., 2003; Pichersky and Raguso, 2018; Singh et al., 2025). This interpretation aligns with the known metabolic shift from growth-related processes toward defense- and quality-related secondary metabolism following topping in tobacco (Zhao et al., 2018; Shi et al., 2025). Yang et al. also reported dynamic changes in phenylpropanoids, sugars, and other quality-related metabolites during the maturation of upper tobacco leaves (Yang et al., 2025a). Therefore, the accumulation of flavonoid-, lignan-, phenolic-, and terpenoid-related differential metabolites in DF may reflect stronger secondary-metabolic remodeling during late maturation rather than a nonspecific enrichment signal alone.

In contrast, differential metabolites in WN were enriched not only in flavonoid-related pathways but also more strongly in starch and sucrose metabolism, glutathione metabolism, and ABC transporters, indicating stronger regulation of sugar metabolism, material transport, and antioxidant homeostasis during late maturation in WN (**Figures 8**, **10**, **11**). In particular, the upregulation of sugars and sugar alcohols such as trehalose, cellobiose, and maltitol suggests that WN leaves may place greater emphasis on osmotic adjustment and storage-related metabolism at late maturation. The environmental correlation analysis further suggested that high-altitude ecological characteristics in WN, such as stronger radiation-related conditions and temperature-related differences, may contribute to flavonoid- and glutathione-related antioxidant responses. These ecological interpretations remain correlative, but they provide a more explicit link between regional environmental conditions and metabolic remodeling. Therefore, tobacco leaves from the two regions may emphasize different metabolic priorities during maturation: DF tends to show stronger dynamic accumulation of metabolites associated with aroma precursors and secondary metabolism, whereas WN places greater emphasis on sugar-metabolism regulation, sugar transport, and antioxidant homeostasis.

### 4.4 Potential relationships between differential metabolites and post-curing style characteristics

Analysis of representative differential metabolites indicated that upregulated vanillin, sinapic acid, pinoresinol, and some flavonoid-, lignan-, and terpenoid-related metabolites in DF during the late maturity stage may be associated with sweet aroma perception, aroma richness, and the potential for subsequent transformation during curing (**Figures 9A**, **11**). The metabolites detected in cured leaves do not directly prove causal determinants of final aroma; rather, they represent post-curing associated markers whose abundance may reflect both precursor accumulation during field maturation and biochemical or chemical transformations during curing. For example, phenylpropanoid- and lignin-related compounds may contribute to phenolic and aromatic aldehyde derivatives, while glycosidically bound aroma compounds stored in tobacco tissues may serve as aroma reservoirs and influence the release of volatile aroma compounds during curing (Cai et al., 2013). Previous studies have shown that phenylpropanoid-, flavonoid-, and carotenoid-related metabolic changes are closely associated with the formation of tobacco aroma precursors, while monoterpenes, sesquiterpenes, and their derivatives can influence odor quality and aroma-style expression (El Tamer et al., 2003; Gerjets et al., 2007; Pichersky and Raguso, 2018).

In WN, the upregulation of sugars and sugar alcohols such as trehalose, cellobiose, and maltitol may be related to sweetness-related perception and to the formation of relevant aroma substrates during curing (**Figure 9B**). Trehalose and other soluble carbohydrates may be hydrolyzed or transformed during curing and can contribute reducing-sugar substrates for Maillard-type reactions, thereby affecting sweet, roasted, or clean aroma notes. Meanwhile, changes in some flavonoid-, phenolic-, and antioxidant-related metabolites in WN may contribute to the maintenance of aroma purity and stability during processing (Ferreyra et al., 2021). Overall, this study revealed potential associations between maturity-related metabolic differences and post-curing style characteristics. However, their specific roles in style formation still need to be further verified by volatile-compound analysis, targeted absolute quantification, curing-stage time-course metabolomics, and multi-environment validation. It should be noted that RT-qPCR validation was performed only in fresh leaves because these samples better represent active transcriptional regulation during field maturation. Although metabolomic differences were evaluated in cured leaves, gene-expression analysis of cured leaves was not used because cured tissues have undergone dehydration, heat treatment, and extensive biochemical transformation and therefore may not reliably reflect pathway-level transcriptional activity.

## 5 Conclusions

By integrating phenotypic observations, color parameters, SPAD values, key enzyme activities, sensory evaluation, widely targeted metabolomics, environmental correlation analysis, and RT-qPCR validation, this study compared differences in leaf maturation between DF and WN, two representative ecological regions in Guizhou, using middle leaves of Yunyan 87. The results showed that, with delayed harvest, tobacco leaves in both regions exhibited typical maturity-associated phenotypes, but maturation progressed more rapidly in DF and more slowly in WN. SPAD values generally declined, whereas POD, LOX, PPO, and SPS activities increased in both regions, indicating continuous physiological and biochemical transitions during leaf maturation. Metabolomic analysis of cured leaves further revealed clear regional differences in harvest-stage-associated post-curing metabolic profiles. Differential metabolites in DF were characterized by relatively high proportions of flavonoids, alkaloids, terpenoids, and other phenolic-related metabolites, together with enrichment of flavonoid biosynthesis and secondary metabolite biosynthesis pathways, whereas WN showed stronger signatures in sugars and derivatives, phenolic compounds, starch and sucrose metabolism, glutathione metabolism, and ABC transporters. Environmental correlation and fresh-leaf RT-qPCR results further suggested that ecological gradients and pathway-level transcriptional divergence during field maturation may contribute to the regional differences in post-curing metabolite profiles. Overall, these findings provide preliminary physiological and metabolic evidence for understanding region-specific maturation and offer a reference for subsequent optimization of harvest timing and style-oriented production of flue-cured tobacco in Guizhou.

## Data Availability

The original contributions presented in the study are included in the article/**Supplementary Material**. Further inquiries can be directed to the corresponding authors.

## Supplementary material

Supplementary Table 1 Meteorological and soil physicochemical variables of the Dafang (DF) and Weining (WN) experimental sites during the 2024 growing season. Values include meteorological and soil physicochemical variables used for environmental association analysis. DF represents the Dafang experimental site, corresponding to the honey-sweet-style tobacco region, and WN represents the Weining experimental site, corresponding to the fresh-sweet-style tobacco region. Solar radiation was used as an approximate indicator of light exposure because direct UV-B measurements were not available for all batches.

Supplementary Table 2 Primer sequences used for RT-qPCR validation of genes related to flavonoid biosynthesis and sugar metabolism/transport.

Supplementary Table 3 List of differential metabolites identified in cured tobacco leaves between two harvest stages in Dafang (DF) and Weining (WN). Differential metabolites were identified separately in Dafang (DF) and Weining (WN) based on comparisons between the two sampling time points in each region. Metabolites were screened according to the differential analysis criteria described in the Methods section. “Up” and “Down” indicate that the metabolite abundance was increased or decreased, respectively, in the later sampling time point relative to the earlier sampling time point.

Supplementary Table 4 Summary of the biological classification of differential metabolites identified in cured tobacco leaves from Dafang (DF) and Weining (WN). Differential metabolites identified in **Supplementary Table 3** were manually reassigned into harmonized chemical categories to enable direct comparison between Dafang (DF) and Weining (WN). Each metabolite was assigned to one unified category only. Percentages were calculated based on the total number of differential metabolites in each region (DF, n = 72; WN, n = 51).
